# Curcumin and Its Derivatives Induce Apoptosis in Human Cancer Cells by Mobilizing and Redox Cycling Genomic Copper Ions

**DOI:** 10.3390/molecules27217410

**Published:** 2022-11-01

**Authors:** Mohammed Ahmed Ismail Alhasawi, Mohammad Aatif, Ghazala Muteeb, Mir Waqas Alam, Mohamed El Oirdi, Mohd Farhan

**Affiliations:** 1 Department of Medical Education, College of Medicine, King Faisal University, Al-Ahsa 31982, Saudi Arabia; 2 Department of Public Health, College of Applied Medical Sciences, King Faisal University, Al-Ahsa 31982, Saudi Arabia; 3 Department of Nursing, College of Applied Medical Sciences, King Faisal University, Al-Ahsa 31982, Saudi Arabia; 4 Department of Physics, College of Science, King Faisal University, Al-Ahsa 31982, Saudi Arabia; 5 Department of Basic Sciences, Preparatory Year Deanship, King Faisal University, Al-Ahsa 31982, Saudi Arabia

**Keywords:** curcuminoids, copper, ROS, pro-oxidant, anticancer

## Abstract

Turmeric spice contains curcuminoids, which are polyphenolic compounds found in the Curcuma longa plant’s rhizome. This class of molecules includes curcumin, demethoxycurcumin, and bisdemethoxycurcumin. Using prostate cancer cell lines PC3, LNCaP, DU145, and C42B, we show that curcuminoids inhibit cell proliferation (measured by MTT assay) and induce apoptosis-like cell death (measured by DNA/histone ELISA). A copper chelator (neocuproine) and reactive oxygen species scavengers (thiourea for hydroxyl radical, superoxide dismutase for superoxide anion, and catalase for hydrogen peroxide) significantly inhibit this reaction, thus demonstrating that intracellular copper reacts with curcuminoids in cancer cells to cause DNA damage via ROS generation. We further show that copper-supplemented media sensitize normal breast epithelial cells (MCF-10A) to curcumin-mediated growth inhibition, as determined by decreased cell proliferation. Copper supplementation results in increased expression of copper transporters CTR1 and ATP7A in MCF-10A cells, which is attenuated by the addition of curcumin in the medium. We propose that the copper-mediated, ROS-induced mechanism of selective cell death of cancer cells may in part explain the anticancer effects of curcuminoids.

## 1. Introduction

Curcumin (C), demethoxycurcumin (dmC), and bisdemethoxycurcumin (bdmC), all of which are classified as curcuminoids, are the main bioactive polyphenols present in the turmeric plant *Curcuma longa* [1,2]. In traditional medical practices all across the world, turmeric has been used for a very long time. Additionally, it is utilized on a large scale as a food preservative, dye, and colorant. Since it was discovered that curcumin had anti-inflammatory [3,4], anticancer [5,6], and antioxidant effects [7,8,9], there has been a significant increase in interest in this compound over the past several years. Even when administered in large amounts, it does not appear to be harmful to either animals [10] or people [11]. From a toxicological point of view, it is considered to be rather harmless. Both in vitro and in vivo [12,13] research has shown that curcumin can prevent the production of potentially mutagenic DNA adducts that are produced from benzo(a)pyrene. It is believed that the majority of curcumin’s biological effects are caused by the antioxidant and radical scavenging characteristics that it possesses [14,15]. 

Attracting our attention are several naturally occurring antioxidants and their modes of action on cellular DNA. Recent studies [16,17,18,19] have shown that plant polyphenols such as curcumin (found in turmeric), resveratrol (found in red grapes and red wine), epigallocatechin-3-gallate (found in green tea), and delphinidin (found in pomegranate juice) induce apoptosis in numerous cancer cell lines. Several of these polyphenols induce apoptotic cell death in various cell lines, but not in healthy cells [16,17,18,19]. This is of special significance because apoptotic cell death is not observed in healthy cells. Numerous putative biological antioxidants, both of plant and animal origin, such as uric acid [20], flavonoids [21], catechins [22], and tannic acid [23], are capable of generating oxygen radicals either on their own or in the presence of transition metal ions, according to previous laboratory research. In the past, we have established that curcumin, when coupled with Cu(II), increases DNA damage through the generation of ROS [24].

Copper is an important metal ion found in chromatin and is closely associated with DNA bases, particularly guanine [25,26]. It is one of the most redox-active metal ions found within living cells. Blood, tissue, and cellular copper levels are significantly elevated in cancer patients, according to multiple studies [27,28]. This is an interesting finding. The rise of copper in the tumor is not dependent on the tissue type, but rather represents a metabolic property of the tumor itself [29]. What remains unresolved is whether this systemic copper increase is a cause or a consequence of the malignant transformation.

As discussed above, the majority of the pharmacological effects of plant polyphenols are believed to result from their ability to scavenge oxygen radicals produced endogenously. However, the antioxidant properties of polyphenolic substances may not fully explain their chemopreventive benefits [30,31]. The majority of plant polyphenols have both antioxidant and pro-oxidant properties [16,17,18,19,32,33], and we have previously hypothesized that the pro-oxidant action of polyphenolics may be a significant factor in their anticancer and apoptosis-inducing effects [31,34]. The pro-oxidant effect generated by the mobilization of endogenous copper ions may be a putative mechanism by which these polyphenols kill cancer cells.

**Figure 1:**
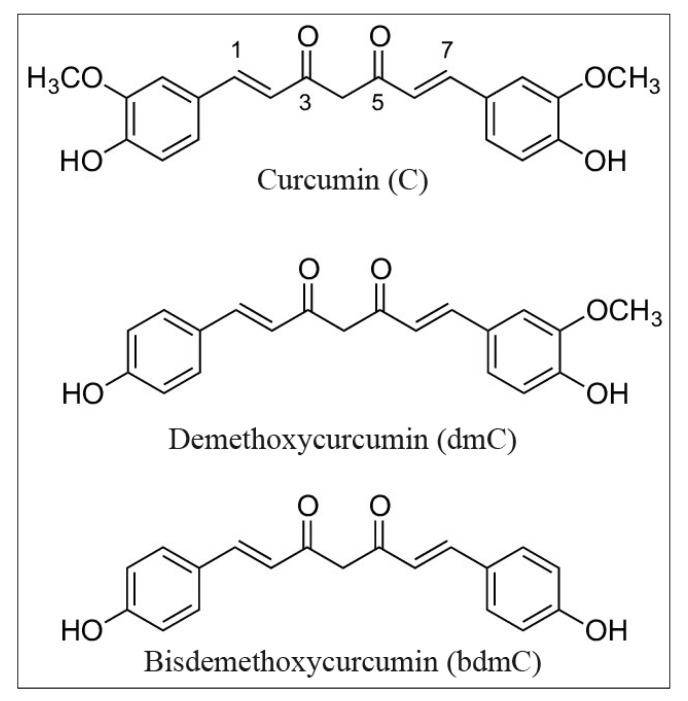
Chemical structure of curcumin, demethoxycurcumin, and bisdemethoxycurcumin.

Curcumin and its two naturally occurring derivatives demethoxycurcumin and bisdemethoxycurcumin have a strong potential to induce oxidative DNA damage, as shown in this study. We demonstrate that these properties of curcumin in cancer cells are reliant on the bioavailability and redox recycling of copper within the cell. The structures of the curcumins utilized in these studies are depicted in Figure 1.

## 2. Materials and Methods

Curcumin, demethoxycurcumin, bisdemethoxycurcumin, DMSO, agarose (normal melting and low melting), Ca^2+^- and Mg^2+^-free phosphate-buffered saline, RPMI 1640 media, Histopaque 1077, metal chelators (neocuproine (Neo), bathocuproine disulphonic acid (Batho), desferrioxamine mesylate (DM) and histidine (His)), and cupric chloride were purchased from Sigma Chemical Co. (St. Louis, MO, USA). All other chemicals were commercial products of analytical grade.

### 2.1. Preparation of Stock Solutions of Curcumins

C, dmC, and bdmC were dissolved in DMSO prior to use as a 3 mM stock solution. Upon addition to reaction mixtures, in the presence of buffers and at the used concentrations, the compounds remained in solution and did not cause a significant pH shift. To examine the effect of solvent on DNA fragmentation, 2% (*v*/*v*) DMSO solution was given to the cells, which was the maximum concentration of DMSO employed in the test compound-treated reaction media. No difference was found in the presence or absence of DMSO, showing that the DMSO doses examined had no effect on the results.

### 2.2. Isolation of Lymphocytes

For each experiment, fresh heparinized blood samples (2.0 mL) from non-smoking healthy donors (authors Mohd Farhan and Mohammad Aatif) were acquired through venipuncture and diluted in Ca^2+^- and Mg^2+^-free PBS. Blood lymphocytes were extracted using Histopaque 1077 (Sigma Diagnostics, St. Louis, MO, USA), and the resulting cells were suspended in RPMI 1640.

### 2.3. Viability Assessment of Lymphocytes

Using the Trypan blue exclusion test [35], the lymphocytes’ viability was assessed prior to and following the reaction. More than 94% of the cells were found to be viable.

### 2.4. Alkaline Single-Cell Gel Electrophoresis (Comet Assay)

The treatment of whole lymphocytes with C, dmC, and bdmC and the subsequent comet assay were carried out as previously described [36,37]. The treatment of cells with C, dmC, and bdmC was performed on slides instead of microcentrifuge tubes so that DNA breakage could be compared to that of lymphocyte nuclei.

### 2.5. Cell Lines and Reagents

The immortalized non-transformed breast cell line MCF-10A and the prostate cancer cell lines PC3, LNCaP, DU145, and C42B were obtained from ATCC (Manassas, VA, USA) and maintained in RPMI 1640 (Invitrogen, Carlsbad, CA, USA). The medium was supplemented with 10% fetal bovine serum (FBS), 100 units/mL penicillin, and 100 µg/mL streptomycin. All cells were cultured in a 5% CO_2_ humidified atmosphere at 37 °C. Stock solutions of C, dmC, and bdmC (50 mM) were created in DMSO, and small aliquots were stored at −20 °C. The stock solutions of different chelators of the metal ions—neocuproine/desferoxamine mesylate/histidine—were created in PBS at a final concentration of 50 mM and were always created fresh prior to experiments. A normal breast epithelial cell line, MCF-10A, was propagated in DMEM/F12 (Invitrogen, Carlsbad, CA, USA) supplemented with 5% horse serum, 20 ng/mL EGF, 0.5 µg/mL hydrocortisone, 0.1 µg/mL cholera poison, 10 µg/mL insulin, 100 units/mL penicillin, and 100 µg/mL streptomycin in a 5% CO_2_ climate at 37 °C.

MCF-10A+Cu cells are MCF-10A cells that were cultured in their normal culture media (above) with extra supplementation of 25 µM CuCl_2_ for a month.

### 2.6. Cell Growth Inhibition Studies by 3-(4,5-Dimethylthiazol-2-yl)-2,5 Diphenyltetra-Zolium (MTT) Assay

In 96-well microtiter plates, 2 × 10^3^ cells were seeded per well. Following an overnight incubation, the standard growth medium was replaced with a new medium containing varied concentrations of curcuminoids diluted from a 50 mM stock. As described in each investigation, various chelators were added to appropriate assays. After 3 days of incubation, 25 µL of MTT solution (5 mg/mL in PBS) was added to each well, and the plates were incubated at 37 °C and 5% CO_2_ for an additional 2 h.

After completion of the 2 h incubation, the supernatant was removed and MTT formazan, framed by metabolically viable cells, was broken down in DMSO (100 µL) by blending for 30 min on a gyratory shaker. The absorbance was estimated at 595 nm on an Ultra Multifunctional Microplate Reader (TECAN, Durham, NC, USA). Every treatment had eight replicate wells, and the measure of DMSO in the response blend never surpassed 0.1%. Additionally, each examination was repeated at least three times. 

### 2.7. Detection of Apoptosis Using Histone/DNA ELISA

The Cell Death Detection ELISA Kit (Roche, Palo Alto, CA, USA) was utilized to identify apoptosis in growth cells treated with variouscurcuminoids. Cells were treated with curcuminoid mixes or DMSO control for 72 h. After treatment, the cytoplasmic histones and DNA from cells were isolated and incubated in microtiter plate modules covered with anti-histone antibody. The peroxidase-conjugated anti-DNA antibody was utilized for the immobilized histone/DNA followed by color advancement with ABTS substrate for peroxidase. The spectrophotometric absorbance of the examples was read by using an Ultra Multifunctional Microplate Reader (TECAN, Durham, NC, USA) at 405 nm.

Reactions were additionally performed with particular metal ion chelators. DM (50 µM) was utilized for the chelation of Fe (II) particles, His (50 µM) was utilized for Zn (II), and Neo (50 µM each) was utilized for the chelation of Cu(II) particles. Free radical scavengers (catalase 20 µg/mL, superoxide dismutase (SOD) 20 µg/mL, and thiourea (TU) 0.1 mM) were utilized to examine the role of ROS in the intracellular reaction of copper with various curcuminoids.

### 2.8. Soft Agar Colonization Assay

In 24-well plates, 4 × 10^5^ cancer cells were seeded in 0.5 mL of culture medium containing 0.3% *w*/*v* top agar layered over 0.7% *w*/*v* basal agar (with culture medium and supplements). The culture was treated with curcumin or DMSO at the time of seeding, with or without a cuprous chelator. After 25 days of culture time, the colonies were counted. There were three replicates of each experiment, and the mean values were presented.

### 2.9. Cell Migration Assay

A cell migration assay was performed by utilizing 24-well transwell permeable supports with 8 mm pores (Corning, NY, USA). Cells were suspended in a serum-free medium and seeded into the transwell embeds. The bottom wells were loaded with media containing complete media. After 24 h, cells were stained with 4 mg/mL calcein AM (Invitrogen, Carlsbad, CA, USA) in PBS at 37 °C for 1 h and detached from inserts by trypsinization. The fluorescence of the migrated cells was read using an Ultra Multifunctional Microplate Reader (TECAN, Durham, NC, USA). The cells were grown in the presence and absence of curcumin (25 µM) with or without neocuproine (50 µM).

### 2.10. Real-Time Reverse Transcriptase PCR

Following the manufacturer’s instructions, total RNA was extracted using TRIzol (Invitrogen) reagent. mRNA expression was quantified using real-time PCR. Sequences of primers (Table 1) for CTR1, ATP7A, and GADPH were the same as reported earlier [38,39], and the amount of RNA was normalized to GAPDH expression.

### 2.11. Small Interfering RNA (siRNA) Transfection

siRNA transfections were carried out as previously described [39]. Santa Cruz Biotechnology, Inc. was contacted for CTR1-specific siRNA. A garbled siRNA was utilized as a control.

Following the manufacturer’s instructions, transfections were carried out with Lipofectamine RNA iMAX Transfection Reagent (Invitrogen); 48 h before the experiment, CTR1 was silenced by siRNA.

### 2.12. Statistical Analysis

The statistical analysis was conducted as outlined by Tice et al. [40] and is expressed as the standard error of the mean (±S.E.M.) for three independent experiments. A Student’s t-test was used to examine statistically significant differences. ANOVA was used to conduct analysis of variance. *p*-values less than ≤ 0.05 were considered statistically significant.

## 3. Results

### 3.1. DNA Breakage by Curcumin in Human Peripheral Lymphocytes

**Figure 2:**
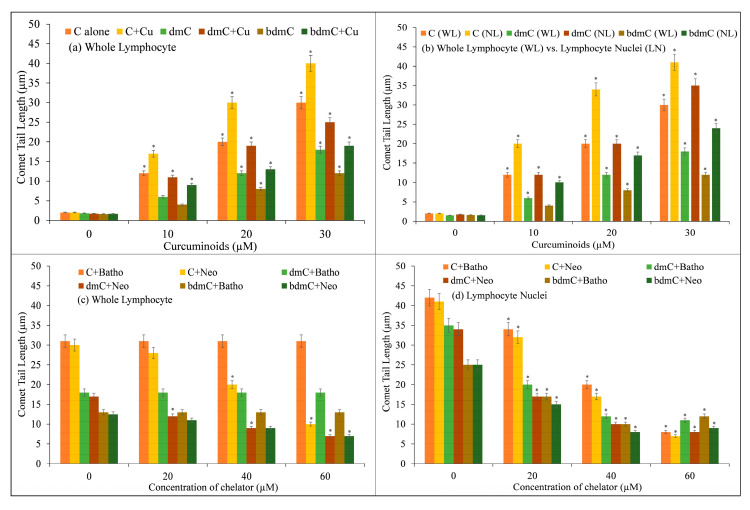
DNA cleavage in human peripheral lymphocytes by C, dmC, and bdmC. (**a**) Lymphocyte cells were treated in microfuge tubes with a reaction mixture for one hour at 4 degrees Celsius in the dark. The reaction mixture consisted of RPMI (400 µL); Ca^2+^- and Mg^2+^-free PBS; and increasing concentrations of C, dmC, and bdmC (0–30 µM), alone and with a fixed concentration of Cu(II) (25 µM), and was further processed for comet assay. (**b**) Comparison of DNA cleavage by C, dmC, and bdmC in whole lymphocytes and lymphocyte nuclei using the comet test. Whole lymphocyte cells/lymphocyte nuclei imbedded in agarose were treated for 1 h at 4 °C with the reaction mixture comprising the appropriate concentrations of C, dmC, and bdmC (0–30 µM) and then subjected to comet assay. Whole lymphocytes (**c**) and lymphocyte nuclei (**d**) embedded in agarose were incubated with the reaction mixture (2.0 mL) containing C, dmC, and bdmC (30 µM) and the indicated concentrations of membrane permeable copper chelator (Neo) or membrane impermeable copper chelator (Batho) at 4 °C for 1 h and then subjected to comet assay. The presented values are the mean ± S.E.M. of three separate experiments. The error bars represent ±S.E.M. * *p* < 0.05 compared to untreated control cells.

Increasing concentrations of C, dmC, and bdmC (0–30 µM) were examined for DNA breakage in isolated human peripheral cells in the presence and absence of CuCl_2_ (25 µM) using the comet assay. Figure 2 depicts the corresponding comet tail length as a function of compound concentration. Despite the fact that C, dmC, and bdmC cause DNA breakage, the degree of DNA breakage is greatly increased in the presence of Cu(II). Cu(II) (25 µM) controls were comparable to untreated lymphocytes in that there was no significant DNA damage. In isolated lymphocytes, the data demonstrate that the curcuminoid–Cu(II) system is capable of DNA damage. It has been found that C causes the most DNA degradation, followed by dmC and bdmC.

In addition, the ability of C, dmC, and bdmC to break DNA in lymphocytes and nuclei was studied. As the membrane and cytoplasmic barrier are abolished in the lysed version of the comet experiment, it is reasonable to presume that the compounds can interact directly with the cell nucleus. Therefore, much more DNA breakage should be observed in the lysed version compared to the normal version, which uses whole lymphocytes. Increasing concentrations of C, dmC, and bdmC (0–30 µM) were examined for their ability to induce DNA breakage in whole lymphocytes (Figure 2b), and the results were similar to those obtained in lymphocyte nuclei. Using the lysed version of the comet assay, the rate of tail formation is greatly increased, demonstrating that curcuminoids can interact directly with the nuclei. In both whole lymphocytes and lymphocyte nuclei, however, the rate of DNA breakage is greatest for C, followed by dmC and bdmC.

DNA damage induced by C, dmC, and bdmC in whole lymphocytes and lymphocyte nuclei was examined in the presence of the Cu(I)-specific chelators Neo and Batho (Figure 2c,d). Incubation of whole lymphocytes with neocuproine (a copper chelator that can permeate the cell membrane) decreased DNA degradation. Neocuproine’s water-soluble, membrane-impermeable equivalent, bathocuproine disulphonate, did not have the same inhibitory effect. This work also confirmed that C, dmC, and bdmC can degrade DNA in cell nuclei and that such DNA degradation is prevented by neocuproine and bathocuproine disulphonate (both of which can permeate the nuclear pore complex), suggesting that copper is involved in this reaction. Within living cells, the most redox-active metal ions are Fe^3+^ and Cu^2+^. Copper and zinc are the most prevalent metal ions in nuclei [41]. Desferrioxamine mesylate (a Fe(II)-specific chelator) and histidine (a zinc-specific chelator) were examined on DNA breakage in whole lymphocytes and lymphocyte nuclei to evaluate if iron and zinc may play a role in curcuminoid-induced DNA breaking. Desferrioxamine mesylate does not protect against curcuminoid-induced DNA breakage in cell nuclei, despite the fact that it suppresses curcuminoid-induced DNA breakage in whole cells (results not shown). Additionally, histidine did not reduce the DNA breakage generated by curcuminoids in whole lymphocytes or lymphocyte nuclei. These results indicate that the DNA damage generated by curcuminoids in lymphocytes requires the mobilization of endogenous copper, presumably chromatin-bound copper, and that Cu(I) is an intermediate in the pathway. Therefore, it is conceivable to establish in vitro and copper-mediated cellular DNA cleavage by antioxidants.

### 3.2. Curcumin Inhibits Growth and Induces Apoptosis in Different Types of Cancer Cells

**Figure 3:**
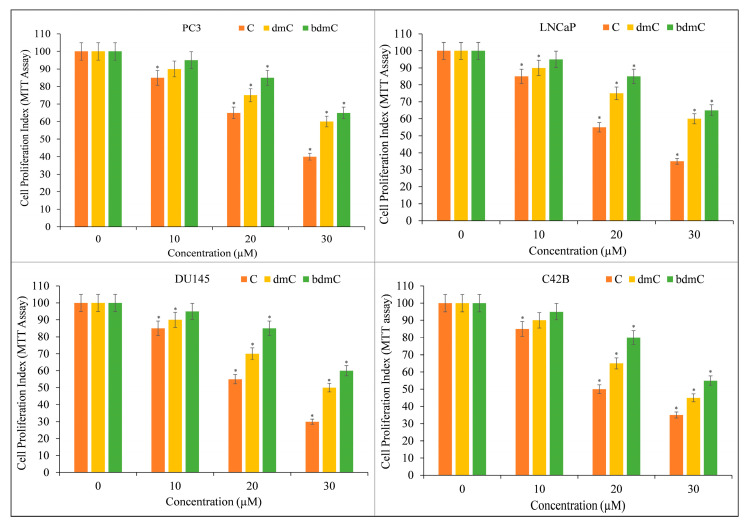
The MTT assay was used to determine the effect of C, dmC, and bdmC on cell proliferation in a variety of prostate cancer cell lines. PC3, LNCaP, DU145, and C42B were treated with the indicated doses of curcuminoids for 72 h. As mentioned in Section 2, the effect on cell growth was identified using an MTT experiment. All results are presented as a percentage of control standard error of triplicate measurements. * *p* < 0.05 versus the corresponding untreated control.

Several prostate cancer cell lines, including PC3, LNCaP, DU145, and C42B, were treated with varying doses of C, dmC, and bdmC. The MTT assay was utilized to investigate whether curcuminoids affected the growth of cancer cells. Cancer cell proliferation was suppressed by all curcuminoids in a concentration-dependent manner (Figure 3). The inhibition was found to be significantly more potent for C compared to dmC and bdmC.

**Figure 4:**
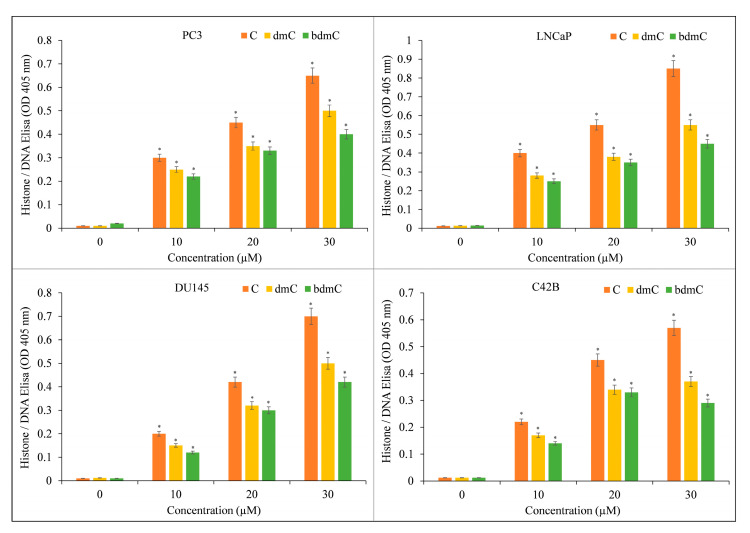
Induction of apoptosis by C, dmC, and bdmC in several cancer cell lines. The Cell Death Detection ELISA Kit (Roche, Palo Alto, CA, USA) was used to detect apoptosis in cancer cell lines after 72 h of incubation with increasing concentrations of curcuminoids, as shown in the figure and detailed in Section 2. The stated values are the ±S.E.M. of three separate experiments. * *p* value < 0.05 in comparison to the control group.

The induction of apoptosis by C, dmC, and bdmC was confirmed using histone/DNA ELISA (Figure 4). C was determined to be the most potent molecule, validating our earlier findings. C was also the most effective inducer of apoptosis, followed by dmC and bdmC. Thus, we concluded that curcuminoids’ cytotoxicity is dose-dependent. C was the most efficient member of this family. Thus, we used only C in our mechanistic studies.

### 3.3. Curcumin-Induced Antiproliferation and Apoptosis in Cancer Cells Are Inhibited by a Cuprous Chelator but Not by Iron and Zinc Chelators

The membrane-permeable copper chelator Neo reduces the polyphenol-induced oxidative cleavage of cellular DNA in lymphocytes (shown above), showing that endogenous copper is involved in the process. As lymphocytes vary from cancer cells, we decided to investigate this finding about curcumin further, and we duplicated this effect in cancer cell lines.

**Figure 5:**
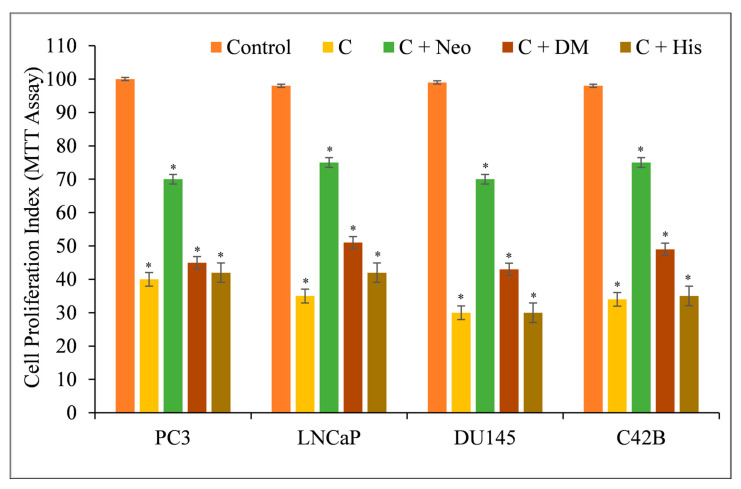
The effects of metal-specific chelators on the antiproliferative activity of curcumin in four distinct prostate cancer cell lines. Cancer cells were treated with 25 µM of curcumin alone or in the presence of copper chelator neocuproine (Neo), iron chelator desferrioxamine mesylate (DM), or zinc chelator histidine (His), as shown in the figure. The metal chelator concentration utilized was 50 µM. After 72 h of treatment, the MTT assay specified in Section 2 was conducted. The values presented are the ±S.E.M. of three separate experiments. * *p* value < 0.05 when compared to the control group.

Therefore, we repeated the experiment with malignant cells and discovered that only the copper chelator Neo substantially protected PC3, LNCaP, DU145, and C42B cells from the growth-inhibiting effects of C (Figure 5). The iron and zinc chelators DM and His were unable to restrict cell growth considerably.

**Figure 6:**
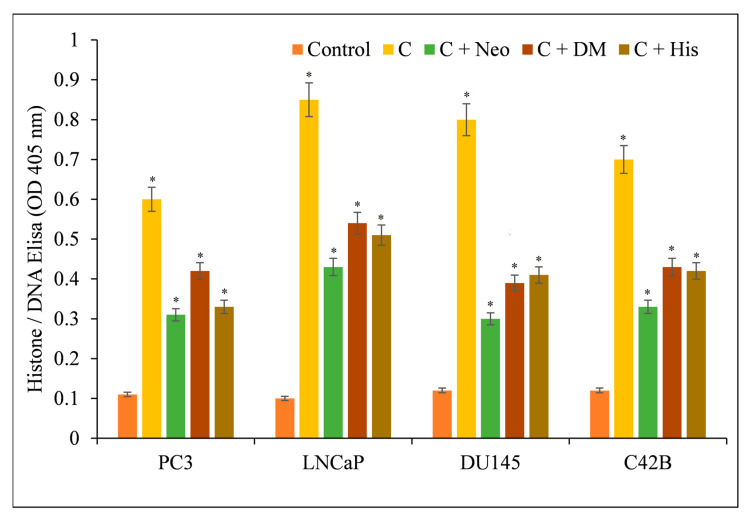
Effects of various metal chelators on the induction of apoptosis by curcumin in four distinct cancer cell lines. As depicted in the image, PC3, LNCaP, DU145, and C42B cancer cells were treated with 25 µM of curcumin alone or in the presence of the copper chelator neocuproine (Neo), the iron chelator desferrioxamine mesylate (DM), or the zinc chelator histidine (His). The concentration of metal chelators utilized was 50 µM. After 72 h of treatment as specified in Section 2, ELISA was performed. The stated values are the ±S.E.M. of three separate experiments. * *p* value < 0.05 in comparison to the control group.

Additionally, the effect of several metal chelators on curcumin-induced apoptosis was evaluated. This protection was not found in the presence of iron or zinc chelators (Figure 6), supporting the conclusion that curcumin’s anticancer mechanism includes the mobilization of endogenous copper.

### 3.4. Curcumin Limits the Cancer Cell Proliferation in a Clonogenic Assay

**Figure 7:**
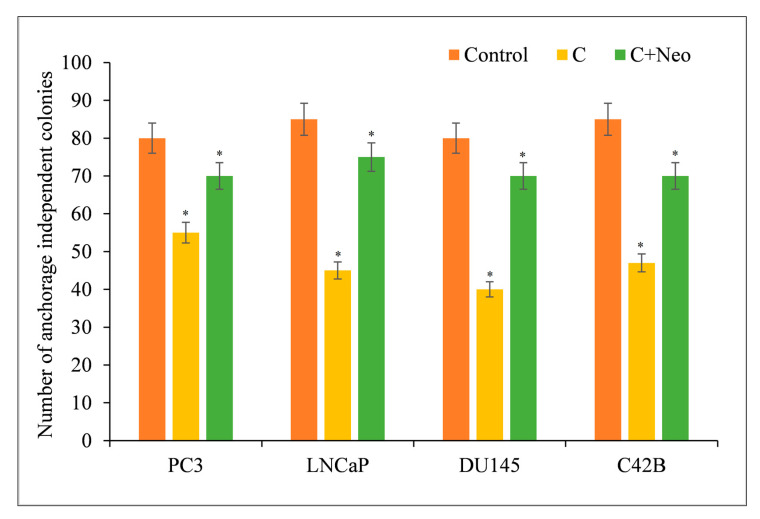
PC3, LNCaP, DU145, and C42B cancer cells (3 × 10^4^) were plated in 24-well plates as described in Section 2. Cultures were supplemented with curcumin at a concentration of 25 µM with or without 50 µM of cuprous chelator (Neo). Experiments were carried out in triplicate and mean values are reported. * *p* < 0.05 when compared to control. Neo alone was also used as a negative control in the experiment. It, however, had no effect on the number of anchorage-independent colonies of cancer cells.

The clonogenic (colony formation) assay is an in vitro cell survival assay dependent on a single cell’s ability to form a colony. Curcumin reduced the frequency of anchorage-independent colonies in cancer cells (Figure 7). However, the impact of curcumin was neutralized by the presence of the cuprous chelator neocuproine. This was similar to the findings involving curcumin and copper chelators discussed before.

### 3.5. Apoptosis of Cancer Cells Induced by Curcumin Is Mediated by ROS

ROS are produced when Cu(I) is reoxidized to Cu(II) in the presence of molecular oxygen [18,19]. To investigate this hypothesis, the effect of several ROS scavengers, including superoxide dismutase, catalase, and thiourea, on curcumin-induced cytotoxicity against four unique prostate cancer cell lines was examined. Thiourea eliminates hydroxyl radicals, while superoxide dismutase and catalase eliminate superoxide anions and hydrogen peroxide. As shown in Table 2, all three scavengers inhibited the apoptotic activity elicited by curcumin in all cancer cell lines. The production of ROS is responsible for the curcumin-induced cell death. Generation of superoxide anions may spontaneously result in the synthesis of H_2_O_2_, which in turn results in the formation of hydroxyl radicals via oxidation of reduced copper (Fenton reaction). Compared to normal cells, most cancer cells contain an imbalance of antioxidant enzymes. ROS levels can overwhelm the antioxidant capability of cancer cells, leading to permanent damage and death [42,43].

Along with curcumin, cancer cells were treated with several ROS scavengers, including TU, 700 µM Thiourea; Cat, 100 µg/mL catalase; and SOD, 100 µg/mL superoxide dismutase. The effect on apoptosis was evaluated using histone/DNA ELISA as described in Section 2. The stated values are the ±S.E.M. of three separate experiments. “Apoptosis (folds)” is the fold increase in apoptosis compared to untreated control.

### 3.6. Copper Chelation Reverses Curcumin-Inhibited Migration of Cancerous Cells

**Figure 8:**
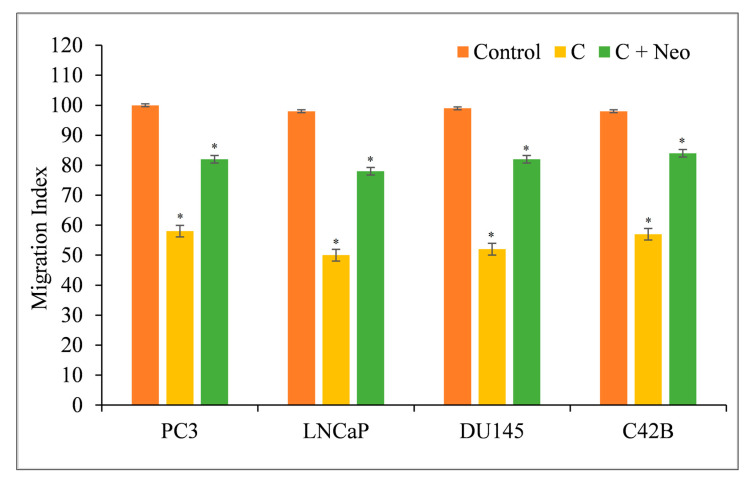
Curcumin’s effect on the migration of PC3, LNCaP, DU145, and C42B cancer cells in the presence of the copper chelator neocuproine. As mentioned in Section 2, a cell migration test was performed utilizing 24-well transwell permeable supports with 8 mm pores (Corning, NY, USA). In the presence and absence of curcumin (25 µM) and neocuproine (50 µM), the cells were developed. On an Ultra Multifunctional Microplate Reader, the fluorescence of the migrating cells was measured (TECAN, Durham, NC, USA). The stated values are the ±S.E.M. of three separate experiments. * *p* value < 0.05 in comparison to the control group.

Metastasis is characterized by the migration and invasion of malignant cells into secondary sites [18,19]. Curcumin inhibited the migratory capacity of PC3, LNCaP, DU145, and C42B cells, making them less likely to metastasize (Figure 8). When the membrane-permeable copper chelator Neo was used to remove copper from the cells in the presence of curcumin, the cells regained their metastatic potential (Figure 8).

### 3.7. Copper Supplementation Increases the Sensitivity of Normal Breast Epithelial Cells to the Antiproliferative Effects of Curcumin

**Figure 9:**
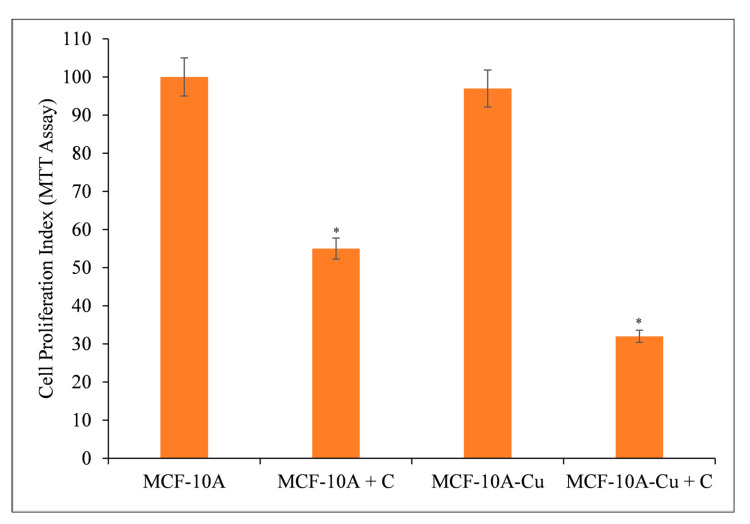
Curcumin’s effect on the inhibition of cell growth in MCF-10A (normal breast epithelial cells) and MCF-10A cells cultivated in Cu(II)-supplemented medium (MCF-10A+Cu). Curcumin (25 µM) was applied to MCF-10A and MCF-10A+Cu (normal cells grown in a medium containing 25 µM CuCl_2_) at the amounts depicted in the figure for 72 h. Subsequently, cell proliferation was measured using the MTT test as indicated in Section 2. The stated values are ±S.E.M. from three different experiments. * *p* value < 0.05 in comparison to the control group.

Normal (non-cancerous) MCF-10A breast epithelial cells were cultured in 25 µM copper-containing media. Curcumin significantly inhibited the growth of copper-supplemented MCF-10A cells (MCF-10A+Cu) compared to non-copper-supplemented MCF-10A cells (Figure 9).

Since malignant transformation is followed by a large increase in intracellular copper levels [18,19,42,43], it is logical to conclude that curcumin’s interaction with intracellular copper is responsible for its prevention of malignant cell proliferation. Exogenous copper sensitizes non-malignant epithelial cells to curcumin-induced cell growth inhibition (Figure 9).

### 3.8. Curcumin Suppresses the Expression of Copper Transporters CTR1 and ATP7A

**Figure 10:**
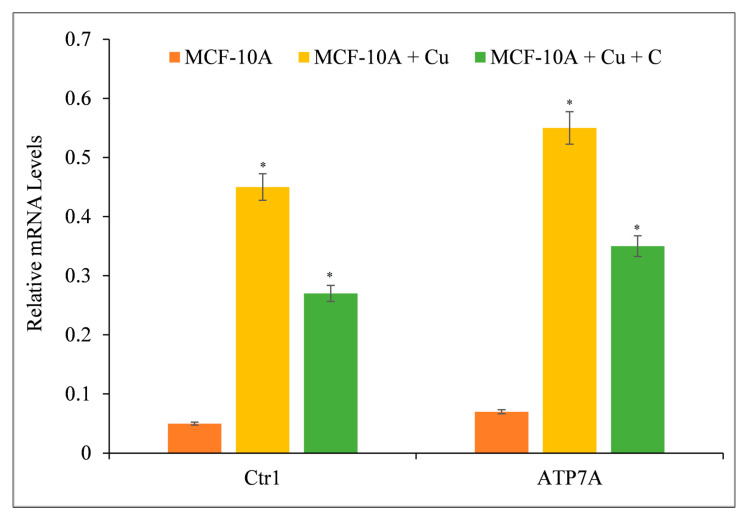
The effect of curcumin on the elevated mRNA transcript levels of copper transporters CTR1 and ATP7A in MCF-10A-Cu cells relative to their parental MCF-10A cells. Total RNA was extracted utilizing TRIzol reagent (Invitrogen, Carlsbad, CA, USA) as directed by the manufacturer. As described in Section 2, CTR1 and ATP7A mRNA expression was quantified using real-time PCR. To determine the effect of curcumin on mRNA expression, only MCF-10A+Cu cells (normal MCF-10A cells grown in a medium containing 25 µM CuCl_2_) with enhanced mRNA expression of copper transporters were treated with 25 µM curcumin. Values reported are ±S.E.M. of three independent experiments. * *p* value < 0.05 when compared to control.

Curcumin reduces growth by interacting with intracellular copper in malignant (Figure 5 and Figure 6) and non-malignant epithelial cells grown in copper-rich media (Figure 9). Since malignant cells express copper transporter CTR1 at a higher level [44], we examined whether copper supplementation promoted copper transporter expression in normal epithelial cells. Copper supplementation in MFC-10A cell growth conditions increased the expression of copper transporters CTR1 and ATP7A significantly (Figure 10) [44,45]. Adding more curcumin to the medium decreased the expression of both copper transporters (Figure 10), indicating that curcumin had an effect on the copper metabolism of cancer cells.

### 3.9. Targeted Silencing of CTR1 in MCF-10A Cells Cultured in Copper-Supplemented Media Reduces Curcumin’s Ability to Inhibit Cell Proliferation

**Figure 11:**
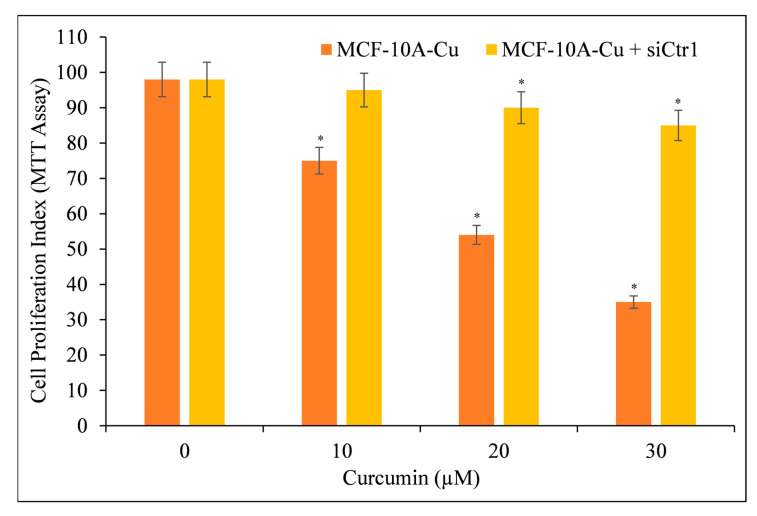
Cell proliferation of MCF-10A+Cu cells (normal MCF-10A cells cultured in a medium containing 25 µM CuCl_2_) was significantly reduced after treatment with curcumin following CTR1 knock-down. MCF-10A+Cu cells were first treated with curcumin or with specific siRNA against CTR1 (siCtr1) for 48 h and then with indicated concentrations of curcumin for 24 h. Values reported are ±S.E.M of three independent experiments. * *p* value < 0.05 when compared to respective control.

We aimed to confirm copper’s essential role in curcumin-mediated growth suppression. As a result, we blocked the copper transporter CTR1 with siRNA (siCtr1) (Figure 11). As previously shown (Figure 10), CTR1 mediates copper uptake in cells, and its expression makes MCF-10A cells more susceptible to curcumin-induced growth suppression. We discovered that blocking copper transporter CTR1 lowered the responsiveness of MCF-10A cells to curcumin in a copper-rich medium.

This proves conclusively that curcumin interacts with cellular copper and that cellular copper is required for curcumin’s anti-proliferative effect on cancer cells.

## 4. Discussion

During the past several years, our laboratory has extensively explored [46,47,48,49] oxidative DNA cleavage processes mediated by polyphenolic antioxidants in the presence of copper ions. The key conclusions obtained from the foregoing findings are as follows: (a) Curcuminoids can cause cellular DNA breakage in lymphocytes in the absence of additional copper ions, likely through the mobilization of endogenous copper ions, resulting in the formation of ROS. (b) Curcuminoids inhibit cell growth in human cancer cell lines in a dose-dependent manner. The polyphenol-induced suppression of cell growth and induction of death in cancer cells is reversed in the presence of the copper chelator neocuproine, demonstrating the function of copper in the cytotoxic effect of antioxidants. (c) Furthermore, copper redox cycling in the presence of curcumin produces ROS, as indicated by a decrease in apoptosis induction in the presence of ROS scavengers, thus offering more evidence and establishing copper as a molecular target for curcumin’s cancer-cell-inhibitory property. (d) C is the most capable of inducing DNA damage in the cancer cell lines evaluated. This is further confirmed by the structure–activity relationship between curcumin and its two naturally occurring derivatives, dmC and bdmC, which confirms that curcumin’s methoxy groups play an important role in anticancer characteristics [50].

The finding that normal breast epithelial MCF-10A cells are resistant to curcumin’s cytotoxic action when compared to tumorigenic prostate cells is intriguing, revealing curcumin’s cancer cell selectivity in exerting its cytotoxic effect. Our observation that MCF-10A cells become sensitive to curcumin-induced cytotoxicity when cultivated in the presence of copper supports the critical role of cellular copper in curcumin-mediated physiological responses that result in cell death.

Copper’s physiological role in malignancies remains poorly understood. Nonetheless, evidence supports the role of increased copper concentrations in tumor angiogenesis [51] and protein aggregation [29]. Experimentally, our hypothesis [31,34] that polyphenols derived from plants interact with intracellular copper and mediate oxidative DNA breakage has been verified [18,19,21,22,42,52]. In this manner, the present investigation gives more support for our notion. Curcumin is also known to target different factors, including NFkB and DYRK2 [53]. In conjunction with these pathways, a pathway driven by ROS may suppress tumor cell proliferation.

When normal epithelial cells were cultivated in the presence of copper, both of the copper transporters evaluated in this work, i.e., CTR1 and ATP7A, were shown to have elevated expression. Curcumin could also inhibit the expression of these transporters. As a result, the expression of copper transporters corresponded with the acquired sensitivity of epithelial cells to curcumin activity. This fact adds another level of regulation to our hypothesis, in which curcumin not only interacts with copper and causes oxidative DNA damage, but also inhibits copper transporters, thereby impeding the copper metabolism of the transformed cells, which appears to be essential for these cells’ survival [28,54].

We were also able to corroborate our findings by inhibiting the expression of the representative copper transporter CTR1 with siRNA. CTR1 silencing destroyed the curcumin sensitivity of MCF-10A cultured with copper supplementation, proving and verifying that copper is required for the selective cell death mediated by curcumin. In summary, the structure of curcumin and the availability of intracellular copper determine curcumin’s ability to trigger oxidative DNA damage in cancer cells. We have provided unique findings that demonstrate the critical involvement of intracellular copper levels, enabled by copper transporters, in the anticancer effect of curcumins in particular and plant-derived polyphenols in general. This adds a new dimension to the design of future mechanism-based research aimed at targeting the tumor microenvironment in order to achieve the desired efficacy of non-toxic anticancer agents such as curcuminoids.

## Figures and Tables

**Table 1:** Primer sets.

Gene Name	Forward Primer	Reverse Primer
CTR1	GCT GGA AGA AGG CAG TGG TA	AAA GAG GAG CAA GAA GGG ATG
ATP7A	ACG AAT GAG CCG TTG GTA GTA	CCT CCT TGT CTT GAA CTG GTG
GADPH	TGG GTG TGA ACC ATG AGA AGT	TGA GTC CTT CCA CGA TAC CAA

**Table 2:** Effect of ROS scavengers on curcumin activity in prostate cancer cell lines.

Cancer Cell Line	Dose	Apoptosis (Folds)	Effect of Scavengers
PC3	Untreated	-	-
	Curcumin (25 µM)	2.54	-
	Thiourea	1.58	37.79
	Catalase	2.03	20.07
	SOD	1.77	30.31
LNCaP	Untreated	-	-
	Curcumin (25 µM)	3.67	-
	Thiourea	1.84	49.86
	Catalase	2.01	45.23
	SOD	1.94	47.13
DU145	Untreated	-	-
	Curcumin (25 µM)	3.46	-
	Thiourea	1.78	48.55
	Catalase	2.13	38.43
	SOD	1.97	43.06
C42B	Untreated	-	-
	Curcumin (25 µM)	3.10	-
	Thiourea	2.17	30.00
	Catalase	2.69	13.22
	SOD	2.51	19.03

## Data Availability

Not applicable.
